# Automatic and Rapid Discrimination of Cotton Genotypes by Near Infrared Spectroscopy and Chemometrics

**DOI:** 10.1155/2012/793468

**Published:** 2012-05-14

**Authors:** Hai-Feng Cui, Zi-Hong Ye, Lu Xu, Xian-Shu Fu, Cui-Wen Fan, Xiao-Ping Yu

**Affiliations:** Zhejiang Provincial Key Laboratory of Biometrology and Inspection and Quarantine, College of Life Sciences, China Jiliang University, Hangzhou 310018, China

## Abstract

This paper reports the application of near infrared (NIR) spectroscopy and pattern recognition methods to rapid and automatic discrimination of the genotypes (parent, transgenic, and parent-transgenic hybrid) of cotton plants. Diffuse reflectance NIR spectra of representative cotton seeds (*n* = 120) and leaves (*n* = 123) were measured in the range of 4000–12000 cm^−1^. A practical problem when developing classification models is the degradation and even breakdown of models caused by outliers. Considering the high-dimensional nature and uncertainty of potential spectral outliers, robust principal component analysis (rPCA) was applied to each separate sample group to detect and exclude outliers. The influence of different data preprocessing methods on model prediction performance was also investigated. The results demonstrate that rPCA can effectively detect outliers and maintain the efficiency of discriminant analysis. Moreover, the classification accuracy can be significantly improved by second-order derivative and standard normal variate (SNV). The best partial least squares discriminant analysis (PLSDA) models obtained total classification accuracy of 100% and 97.6% for seeds and leaves, respectively.

## 1. Introduction

Cotton is an economically important plant grown world-wide as a principal source of staple fiber and vegetable oil. A great deal of effort has been made to improve cotton cultivation and characteristics by genetic engineering [1], such as adapting advantageous varieties to new geographical areas, increasing protein and oil contents of seeds [2], recovering more fertile varieties, and developing disease and insect resistance [3]. Although widely cultivated, transgenic plants have aroused wide concern among the public [4–6], including the transfer of the introduced genes to wild plants and nontransgenic plants, the indirect effects of the transgenic crops on the environment, modification of the biodiversity of wildlife, unpredicted harmful changes in food products, and so on. Therefore, there is an increasing demand for monitoring and verifying the presence and the amount of genetically modified organisms (GMOs) in agricultural crops and in products derived [7, 8].

To perform a transgenic analysis, a primary and basic task is to identify the existence of certain genotype. The currently used methods for transgenic product identification include protein-based methods [9], DNA-based methods [10], microscopy, spectroscopy, and chromatography [1, 11]. The rationale behind NIR transgenic analysis is the spectral absorbance of molecular bonds such as C–H, C–N, and C–O that is related to the phenotypic changes (expression level) caused by genotypic changes. Then, chemometric methods are used to extract detailed information concerning sample genotypes. For transgenic identification, some advantages make NIR spectroscopy a useful alternative tool to biological analytical methods: (1) no or less sample preparation, (2) reduced analysis time and cost, (3) simultaneous characterization of multiple components influenced by genotype, and (4) feasibility of online analysis [12]. However, compared with biological analysis methods, NIR-transgenic analysis also suffers some disadvantages. Firstly, due to the baseline, low signal-to-noise ratio (SNR) and the natural weak absorbance of some components, the sensitivity of NIR analysis is much lower. To increase the analytical sensitivity, proper data-preprocessing methods, such as smoothing [13], taking derivatives [13], and standard normal variate (SNV) [14], are required to remove background, improve SNR, and enhance spectral resolution. Another practical problem is the existence of outliers which would degrade or spoil the classification models. Considering the multivariate nature and uncertainty of potential spectral outliers, it is important to detect and exclude the real outliers before any chemometric models are developed. 

The aim of this paper is to develop a rapid, accurate, and robust method for genotype analysis of cotton plants (parent, transgenic, and parent-transgenic hybrid) by near infrared (NIR) spectroscopy and robust partial least squares discriminant analysis (PLSDA) [15] methods. To tackle the problem of outliers, robust principal component analysis (rPCA) [16] was applied to each separate sample group to detect and exclude the measured outliers. The influence of different data-preprocessing methods on model prediction performance was also investigated.

## 2. Experimental and Methods

### 2.1. Sample Collection and NIR Spectra Acquisition

The cotton plants of three different genotypes including parent, transgenic, and parent-transgenic hybrid were collected from two plantations as shown in Table 1. The transgene was modified with *Bt* toxins inserted into the nuclear genome. All the leave samples were 3-lobed ones from the top of the plants. The collected leaves were cleaned with water and dried at 60°C for 24 hours before grinding. For seed collection, immature and deficient seeds were manually excluded. Both leaves and seeds were then ground into powders and finally sifted through a 0.45 mm sieve.

NIR spectra were collected using a TENSOR37 Fourier Transform NIR spectrometer (Bruker, Ettlingen, Germany) in the wavelength range of 4000–12000 cm^−1^. For each sample, 32 scans were performed with a resolution of 8 cm^−1^ at 25°C using OPUS6.5 software. An increase in scanning time did not significantly improve the signal. The average of the 32 scans was used as a raw spectrum for further data analysis.

### 2.2. Outliers Detection by rPCA

Detection of NIR spectral outliers is far from a trivial task for some reasons. Firstly, NIR spectra are of multivariate nature; for example, a spectrum can have more than one thousand analytical channels, while the size of training set is usually less than 100. Therefore, in the case of “large *p*, small *n*” problem, sufficient description of the multivariate sample distribution usually requires dimension reduction of the measured data by latent-variable methods, such as PCA. Moreover, when performing outlier detection, the masking effects of multiple outliers need to be considered, so robust class models resistant to outliers are required.

 Robust principal component analysis (rPCA) was based on robust estimators of principal components (PCs) and the resulted projection distances and residuals. Hubert et al. [17] proposed an improved version of rPCA algorithm, which was numerically more stable for high-dimensional data and computationally effective. In rPCA, score distance (SD) is defined as the sample distance from the data center in PC space and orthogonal distance (OD) as a measure of the PC projection residual. An object can be classified into one of the following four groups in terms of OD and SD: good PCA-leverage points (with large SD and small OD), orthogonal outliers (with small SD and large OD), bad PCA-leverage points (with large SD and large OD), and regular objects (with small SD and small OD). 

### 2.3. PLSDA

Since PLS is a commonly used method in chemometrics, here only a brief introduction to multiclass PLSDA is presented. The training NIR spectral set can be arranged in an *N* × *p* matrix **X **containing the absorbance measurements at *p* wavelengths for *N* samples. For multiclass problems, *N* denotes the total size of all the *B* (in this paper, *B* = 3) different classes. A response matrix **Y **(*N* × *B*) is constructed containing the category variables of each sample in **X**, where each row vector in **Y** indicates the class of a sample. If a sample belongs to class *i*(*i* = 1 : *B*), then the *i*th element of its response variable is assigned a value of 1 and otherwise 0. Then *B* PLS models can be developed to fit each column of **Y **using** X**. For prediction, an unknown object is classified into class *j*(*j* = 1 : *B*) when the *j*th element of its predicted response vector is the nearest to 1. 

### 2.4. Model Validation and Evaluation

For PLSDA, an important problem is to select the number of latent components or determine the model complexity. Including too many latent variables would lead to an unnecessarily complicated model that tends to overfitting, while selecting too few components would lose useful data information and fail to classify the samples sufficiently. Therefore, an improved cross-validation algorithm, Monte Carlo cross-validation (MCCV) [18], was used for this purpose. By multiple resampling and leaving out a higher percent of training samples for prediction, MCCV has been proved to be a reliable method to estimate model complexity and can reduce the risk of overfitting effectively. The RMSEMCCV (root mean square errors of MCCV) values with different model complexity were calculated and then tested with a well-established *F*-test procedure [19, 20]. To avoid selecting too many latent variables, this *F*-test procedure determines model complexity as obtaining an RMSEMCCV not significantly higher than the lowest RMSEMCCV with least model complexity.

To evaluate the performance of discriminant models, sensitivity and specificity of prediction set for each genotype were calculated as follows:

(1) $$\begin{array}{c} \text{Sens}.=\frac{\text{TP}}{\text{TP}+\text{FN}},\;\;\text{Spec}.=\frac{\text{TN}}{\text{TN}+\text{FP}}, \end{array}$$

where TP, FN, TN, and FP denote the numbers of true positives, false negatives, true negatives, and false positives, respectively.

All the data analysis was performed on MATLAB 7.0.1 (Mathworks, Sherborn, MA).

## 3. Results and Discussion

**Figure 1:**
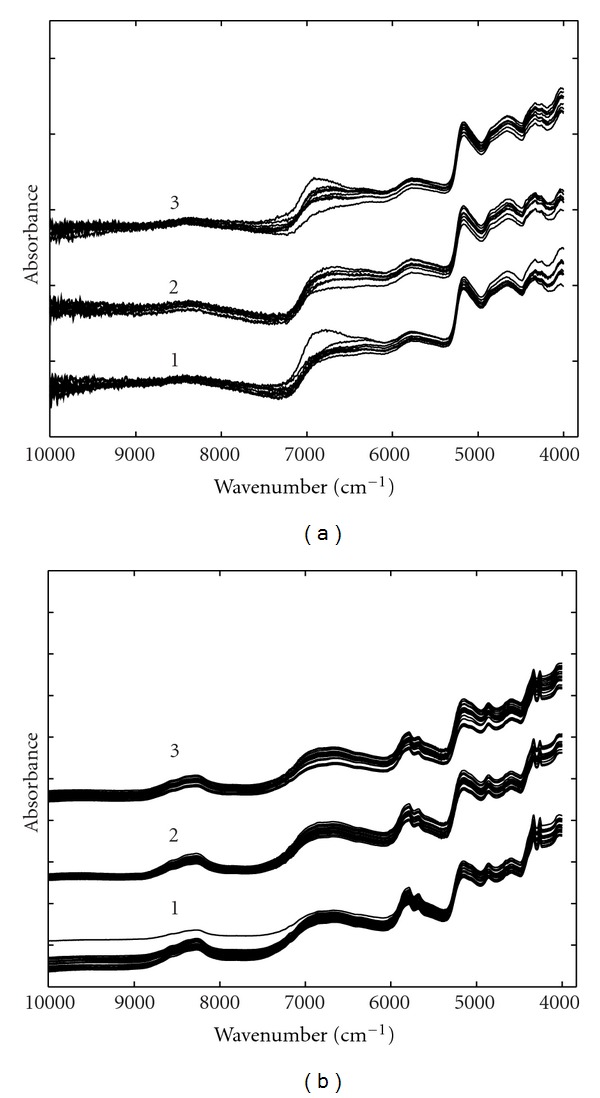
Some of the raw NIR spectra of cotton leaves (a) and seeds (b). The genotypes were (1) parent 222, (2) transgenic 07-19, and (3) hybrid 08-6.

**Figure 2:**
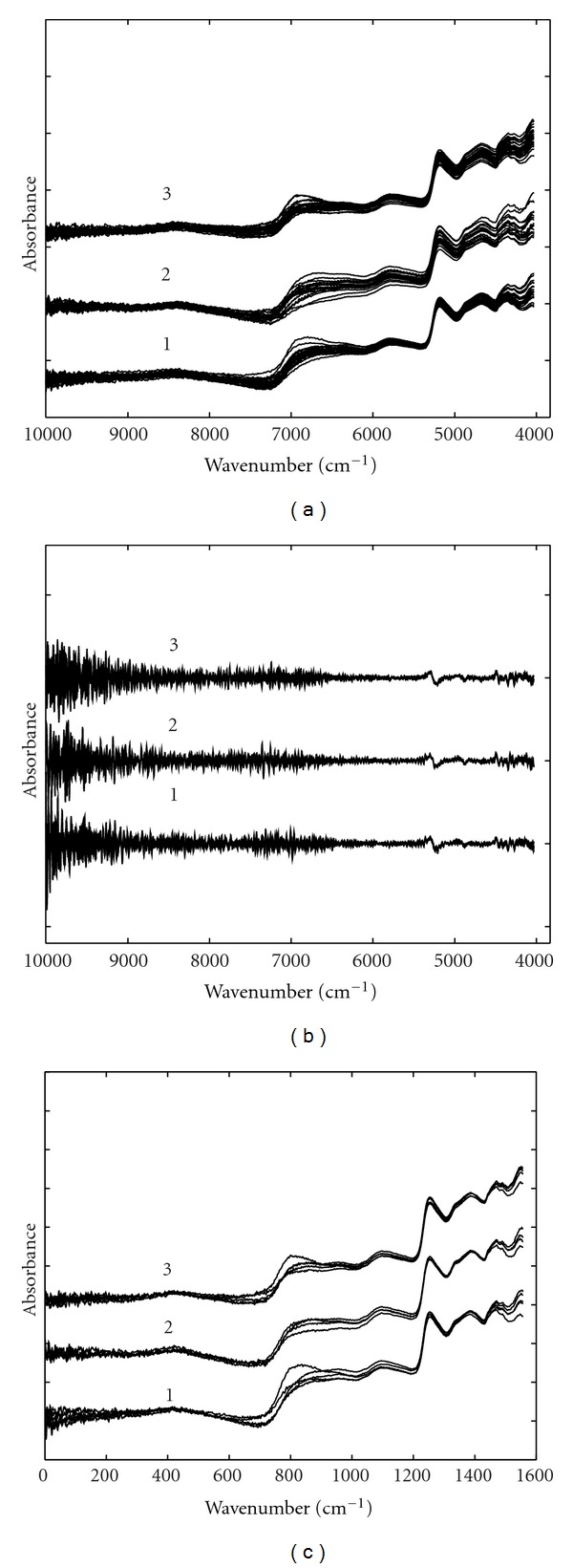
Some of the NIR spectra of cotton leaves preprocessed by (a) smoothing, (b) second-order derivative, and (c) SNV. The genotypes were (1) parent 222, (2) transgenic 07-19, and (3) hybrid 08-6.

**Figure 3:**
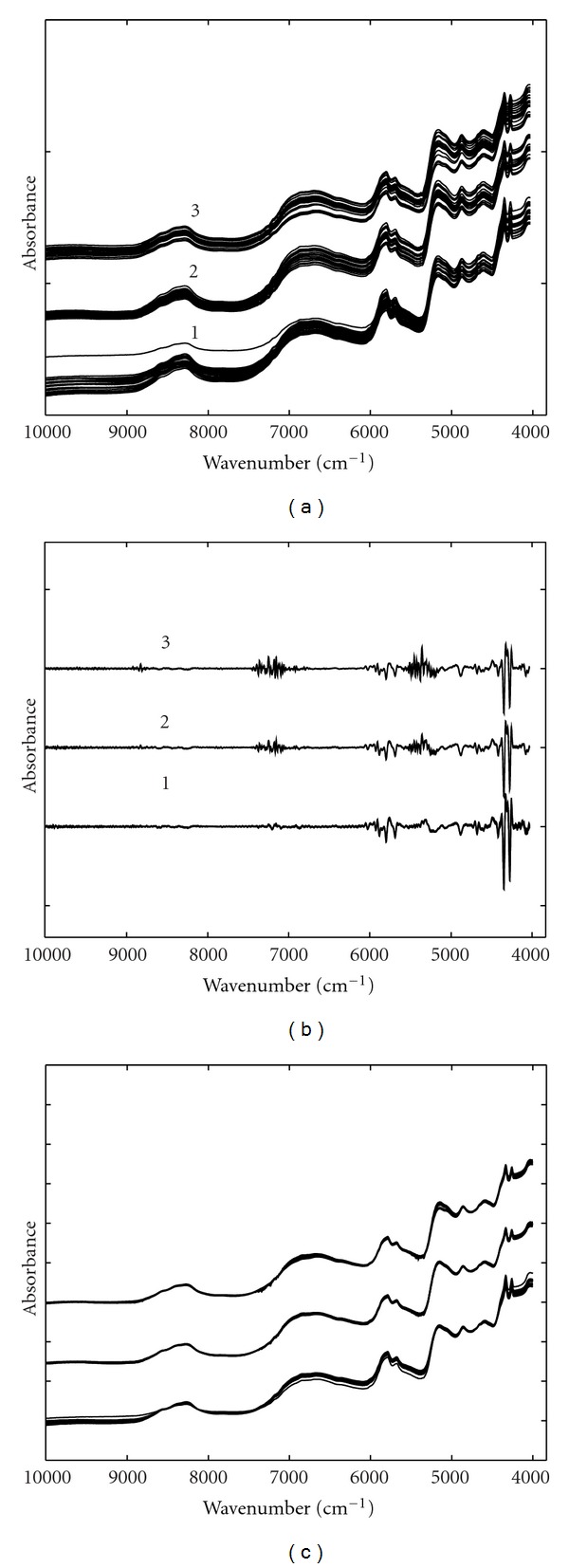
Some of the NIR spectra of cotton seeds preprocessed by (a) smoothing, (b) second-order derivative, and (c) SNV. The genotypes were (1) parent 222, (2) transgenic 07-19, and (3) hybrid 08-6.

Some of the measured spectra of cotton seeds and leaves from three different genotypes are shown in Figure 1. The interval between 12000 cm^−1^ and 10000 cm^−1^ is contaminated with significant noise and was excluded from data analysis. Seen from Figure 1, for both seeds and leaves, the spectra of three genotypes assume very similar absorbance bands and the data are characterized by low absorbance and baseline. Therefore, proper data preprocessing methods were required to reduce various undesirable factors in the raw data. Figures 2 and 3 show the preprocessed spectra obtained by smoothing and taking second-order derivative and SNV transformation for leaves and seeds, respectively. Smoothed spectra seem to have an improved SNR at the cost of losing some detailed information. Second derivative can effectively improve resolution but has a degraded SNR. From Figure 3, it is very obvious the detailed information around 7200 cm^−1^ in second-order derivative spectra is very useful for classification.

**Figure 4:**
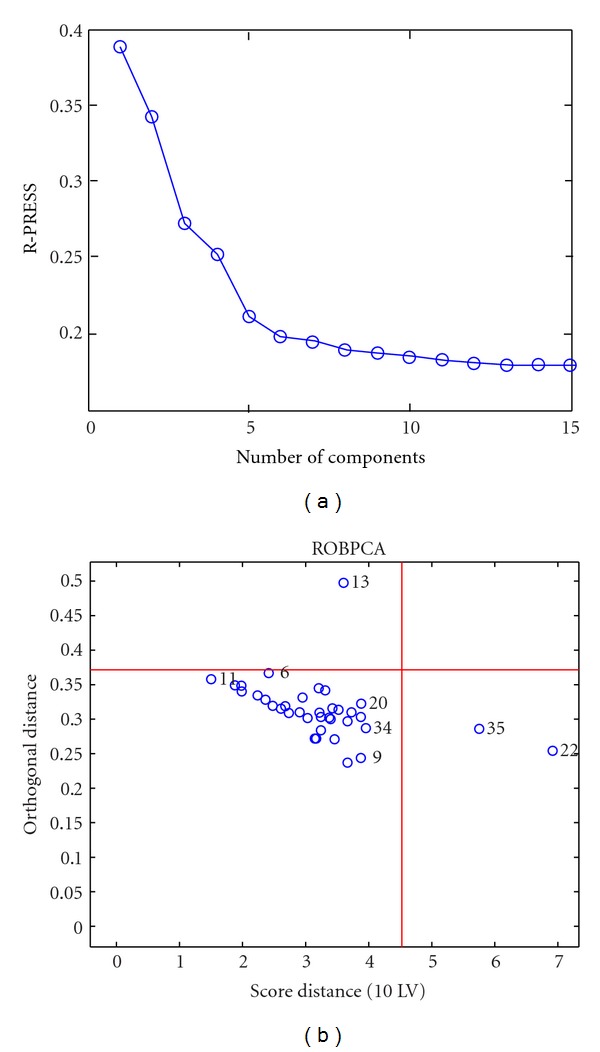
Robust PCA outlier diagnosis of the transgenic cotton leaves based on raw spectra.

Outlier detection was performed based on rPCA of the raw spectral data at a significance level of 0.05. Because each genotype has a different sample distribution, rPCA was performed on each of the genotype. To demonstrate the outlier diagnosis, Figure 4 demonstrates the rPCA plots of the transgenic cotton leaves. 10 components account for 85.77% of the total variances and more components can not decrease the robust cross-validation PRESS (prediction sum of squares) value significantly; therefore, 10 components were selected. Seen from Figure 4(b), sample 13 was detected as orthogonal outliers and samples 22 and 35 as good PCA-leverage points. To select a representative set covering a wide range of samples, only bad PCA-leverage points and orthogonal outliers were excluded and good PCA-leverage samples were retained. The outlier diagnosis results for three genotypes of leaves and seeds are summarized in Table 2.

To select representative training and test sets for model training and validation, K-S algorithm [21] was used to split the samples into a training set and a test set. The K-S algorithm selects the set of training samples that covers the overall sample domain based on their distance (Euclidean distance) from each other. Because the distributions of different genotypes were different, K-S algorithm was performed on each subclass and then the obtained samples were combined to form a training set and test set.  Table 3 demonstrates the splitting of training and test sets with outliers waded.

With different preprocessing methods, PLSDA models were developed. The sampling time of MCCV was 100 and the significance level of the *F*-test was set to be 0.25 as proposed. The prediction results of test set are summarized in Table 4. Seen from Table 4, second derivative and SNV spectra obtained improved prediction accuracy compared with raw and smoothed spectra. For cotton seeds, second-order spectra can correctly classify all the test samples and SNV spectra had just one object wrongly predicted. For leave samples, both second-order derivative and SNV spectra had one sample wrongly predicted. The effects of second-order derivative spectra on classification can be also seen from Figure 3(b), where the three genotypes can be clearly distinguished from the naked eye by some detailed high-frequency information. Compared with the raw data, smoothed spectra cannot improve classification accuracy, which might be attributed to the loss of high-frequency spectral information [22].

## 4. Conclusions

 Rapid and accurate discrimination of three different genotypes of cotton plants were developed by NIR analysis of leaves and seeds. The best models obtained total classification accuracy of 100% and 97.6% for seeds and leaves, respectively. In order to tackle the problem of spectral outliers, robust PCA models were applied to each subclass and were proved to be very effective. SNV and second-order derivative can significantly improve the classification accuracy by removing background and baseline and enhancing resolution. Spectral smoothing can not improve prediction performance due to the possible loss of high-frequency information. The results also demonstrate the removal of background and baseline plays a more important role than enhancing signal SNR for classification.

## Figures and Tables

**Table 1:** Analyzed cotton plants.

Objects	Acquisition time	Plantation	Genotype	Sample size
Seeds	2010.9 2010.9 2010.9	Zhejiang University Zhejiang University Zhejiang University	Parent 222 Transgenic 07-19 Hybrid 08-6	41 40 40

Leaves	2011.10 2011.10 2011.10	China Jiliang University China Jiliang University China Jiliang University	Parent 222 Transgenic 07-19 Hybrid 08-6	41 36 45

**Table 2:** Results of outlier diagnosis.

Objects	Genotype	Orthogonal outliers	Bad PCA leverages	Final data sizes
Seeds	Parent 222 Transgenic 07-19 Hybrid 08-6	16,19 13,21,30,33 14,19,25	1 — —	38 36 37

Leaves	Parent 222 Transgenic 07-19 Hybrid 08-6	34 13 5,29	9 — —	39 35 43

**Table 3:** Splitting of data with outliers waded into training and test sets.

Objects	Genotype	Clean data size	Splitting (training/test)	Total (training/test)
Seeds	Parent 222 Transgenic 07-19 Hybrid 08-6	38 36 37	25/13 25/11 25/12	75/36

Leaves	Parent 222 Transgenic 07-19 Hybrid 08-6	39 35 43	25/14 25/10 25/18	75/42

**Table 4:** Classification results of test set with different preprocessing methods.

Objects	Preprocessing	Wrongly classified	Total accuracy
Seeds	Raw	5	86.1%
	Smoothing	3	91.7%
	2nd derivative	0	100.0%
	SNV	1	97.2%

Leaves	Raw	4	90.5%
	Smoothing	7	83.3%
	2nd derivative	1	97.6%
	SNV	1	97.6%
